# Anxiety, depression and post-traumatic stress symptoms among COVID-19 survivors in Tunis

**DOI:** 10.1192/j.eurpsy.2022.1344

**Published:** 2022-09-01

**Authors:** S. Ajmi, R. Masmoudi, R. Sallemi, I. Feki, J. Masmoudi

**Affiliations:** 1 CHU Hedi Chaker, Psychiatry, sfax, Tunisia; 2 Hospital Hédi Chaker, Sfax, Sfax, Tunisia; 3 Hospital university of HEDI CHAKER, Psychiatry A Department, Sfax, Tunisia

**Keywords:** Depression, post traumatic stress, Anxiety, covid 19

## Abstract

**Introduction:**

In addition to physical problems, patients with COVID-19 suffer from considerable stress throughout the disease crisis and could present psychiatric consequences even after their remission.

**Objectives:**

To assess anxiety, depression and post-traumatic stress symptoms among patients who had recovered from the acute COVID-19 infection in Tunisia.

**Methods:**

A cross-sectional design included 50Tunisian adults who survived COVID-19 virus infection.Participants have been screened with a telephone interview 1 to 3 months after a diagnosis of COVID-19. We used a questionnaire including socio-psychological variables,presence of close relatives being infected, bereavement due to COVID-19 and post infection physical discomforts.The Impact of Event Scale-Revised (IES-R) was used to investigate post-traumatic stress disorder (PTSD). Depression and anxiety were measured using The Hospital Anxiety and Depression Scales (HADS).

**Results:**

The age of the participants ranged from 19 to 86 years.38%were female. Twelve percent (12%) of patients required hospitalization during COVID-19 infection. After a mean of 86.60 days (SD = 23) following the diagnosis, 28 % of patients reported clinically significant PTSD. The rates of depression and anxiety disorders in our population are 20% and 30%, respectively. Seventy percent of patients (70%) reported one or more post infection physical discomforts that the most common symptoms included Difficulty breathing and anosmia. Patients with PTSD, depression or anxiety had a more frequent history of a relative diagnosed positive for corona virus, a longer duration of infection, and more frequently post-infection physical discomfort

**Conclusions:**

Long-term psychological impact of COVID19 should not be ignored and mental health care could play an important role in rehabilitation.

**Disclosure:**

No significant relationships.

